# Predicting *Pseudomonas aeruginosa* drug resistance using artificial intelligence and clinical MALDI-TOF mass spectra

**DOI:** 10.1128/msystems.00789-24

**Published:** 2024-08-16

**Authors:** Hoai-An Nguyen, Anton Y. Peleg, Jiangning Song, Bhavna Antony, Geoffrey I. Webb, Jessica A. Wisniewski, Luke V. Blakeway, Gnei Z. Badoordeen, Ravali Theegala, Helen Zisis, David L. Dowe, Nenad Macesic

**Affiliations:** 1 Department of Infectious Diseases, The Alfred Hospital and School of Translational Medicine, Monash University, Melbourne, Australia; 2 Department of Microbiology, Monash Biomedicine Discovery Institute, Monash University, Melbourne, Australia; 3 Centre to Impact AMR, Monash University, Melbourne, Australia; 4 Department of Biochemistry & Molecular Biology, Monash Biomedicine Discovery Institute, Monash University, Melbourne, Australia; 5 Department of Data Science & AI, Monash University, Melbourne, Australia; Agricultural Biotechnology Research Center, Nankang, Taipei, Taiwan

**Keywords:** antimicrobial resistance, MALDI-TOF MS, machine learning, *Pseudomonas aeruginosa*

## Abstract

Matrix-assisted laser desorption/ionization–time of flight mass spectrometry (MALDI-TOF MS) is widely used in clinical microbiology laboratories for bacterial identification but its use for detection of antimicrobial resistance (AMR) remains limited. Here, we used MALDI-TOF MS with artificial intelligence (AI) approaches to successfully predict AMR in *Pseudomonas aeruginosa*, a priority pathogen with complex AMR mechanisms. The highest performance was achieved for modern β-lactam/β-lactamase inhibitor drugs, namely, ceftazidime/avibactam and ceftolozane/tazobactam. For these drugs, the model demonstrated area under the receiver operating characteristic curve (AUROC) of 0.869 and 0.856, specificity of 0.925 and 0.897, and sensitivity of 0.731 and 0.714, respectively. As part of this work, we developed dynamic binning, a feature engineering technique that effectively reduces the high-dimensional feature set and has wide-ranging applicability to MALDI-TOF MS data. Compared to conventional feature engineering approaches, the dynamic binning method yielded highest performance in 7 of 10 antimicrobials. Moreover, we showcased the efficacy of transfer learning in enhancing the AUROC performance for 8 of 11 antimicrobials. By assessing the contribution of features to the model's prediction, we identified proteins that may contribute to AMR mechanisms. Our findings demonstrate the potential of combining AI with MALDI-TOF MS as a rapid AMR diagnostic tool for *Pseudomonas aeruginosa*.

**IMPORTANCE:**

*Pseudomonas aeruginosa* is a key bacterial pathogen that causes significant global morbidity and mortality. Antimicrobial resistance (AMR) emerges rapidly in *P. aeruginosa* and is driven by complex mechanisms. Drug-resistant *P. aeruginosa* is a major challenge in clinical settings due to limited treatment options. Early detection of AMR can guide antibiotic choices, improve patient outcomes, and avoid unnecessary antibiotic use. Matrix-assisted laser desorption/ionization–time of flight mass spectrometry (MALDI-TOF MS) is widely used for rapid species identification in clinical microbiology. In this study, we repurposed mass spectra generated by MALDI-TOF and used them as inputs for artificial intelligence approaches to successfully predict AMR in *P. aeruginosa* for multiple key antibiotic classes. This work represents an important advance toward using MALDI-TOF as a rapid AMR diagnostic for *P. aeruginosa* in clinical settings.

## INTRODUCTION

*Pseudomonas aeruginosa* is an opportunistic pathogen that causes significant global morbidity and mortality (1), and carbapenem-resistant *P. aeruginosa* has been identified by the World Health Organization (WHO) as a critical priority pathogen (2). The development of antimicrobial resistance (AMR) in *P. aeruginosa* is often due to a complex interplay of intrinsic mechanisms, chromosomal mutations, and the ability to horizontally acquire resistance determinants from other species (3–5). This diverse repertoire can lead to rapid development of AMR to different antimicrobial agents, including last resort treatments for *P. aeruginosa* infections. Furthermore, the global spread of multidrug-resistant, high-risk clones such as ST235, ST111, or ST233 has made treating *P. aeruginosa* increasingly challenging (6). The lack of new antimicrobial discoveries makes optimizing use of current antipseudomonal agents an urgent priority.

Treatment of *P. aeruginosa* infection often begins empirically with later adjustment based on results of antimicrobial susceptibility testing (AST). Accurate and rapid AST methods are thus critical in selecting appropriate antimicrobial agents, both to effectively treat potentially life-threatening infections and to reduce the risk of inducing AMR due to antimicrobial misuse, which can create a selective environment that favors the survival and spread of resistant strains (7). Traditional culture-based AST methods are accurate, but their average turnaround time is approximately 3 days (8), leading to unacceptable delays in treatment.

In the face of these challenges, matrix-assisted laser desorption/ionization–time of flight mass spectrometry (MALDI-TOF MS) has emerged as a key technology in clinical microbiology. The technique characterizes the protein profile of a particular pathogen based on the spectrum recorded mass and quantity of ionized particles. MALDI-TOF MS has already been implemented in clinical microbiology laboratories for routine bacterial identification and investigation is ongoing for its potential use for AST by comparing the spectra generated with known AMR patterns (9). Current approaches for use of MALDI-TOF MS in this setting rely on matches to AMR-related biomarkers. For instance, the presence of a 2,415 *m*/*z* peak has been used to detect PSM-mec expression in methicillin-resistant coagulase-negative staphylococci, yielding a specificity of 1 (10). While this approach can be effective for specific resistance mechanisms, it limits the applicability of MALDI-TOF MS for more complex AMR mechanisms that may result from multiple interacting factors. Recent advancements in machine learning (ML) algorithms and computing resources make artificial intelligence (AI) approaches a promising way to predict AMR using MALDI-TOF MS data in key bacterial pathogens (11–15). This has included *P. aeruginosa* but only with a limited number of tested antimicrobials and not using gold standard AST methodology (16).

To address these gaps, we applied ML approaches to predict AMR in *P. aeruginosa* with broader applicability to other use cases relying on analysis of MALDI-TOF spectra. We present two methods aimed at enhancing the predictive performance. First, MALDI-TOF MS is a high-dimensional data type with *m*/*z* values conventionally ranging from 2,000 Da to 20,000 Da. Training a model with a limited data set may result in a “big-*p*, little-*n*” problem (*p* >> *n*; the number of predictors [*p*] is much larger than the sample size [*n*]) and overfitting. We therefore introduced dynamic binning, a novel and simple feature engineering technique that reduces the feature set while maintaining AMR prediction power compared to conventional approaches. Second, we employed transfer learning to acquire an embedding layer from a large publicly available MALDI-TOF MS data set, then used both dynamic binning features and newly acquired features to develop an ensemble model to predict AMR.

## RESULTS

### Data collection and resistance profiles

**Fig 1:**
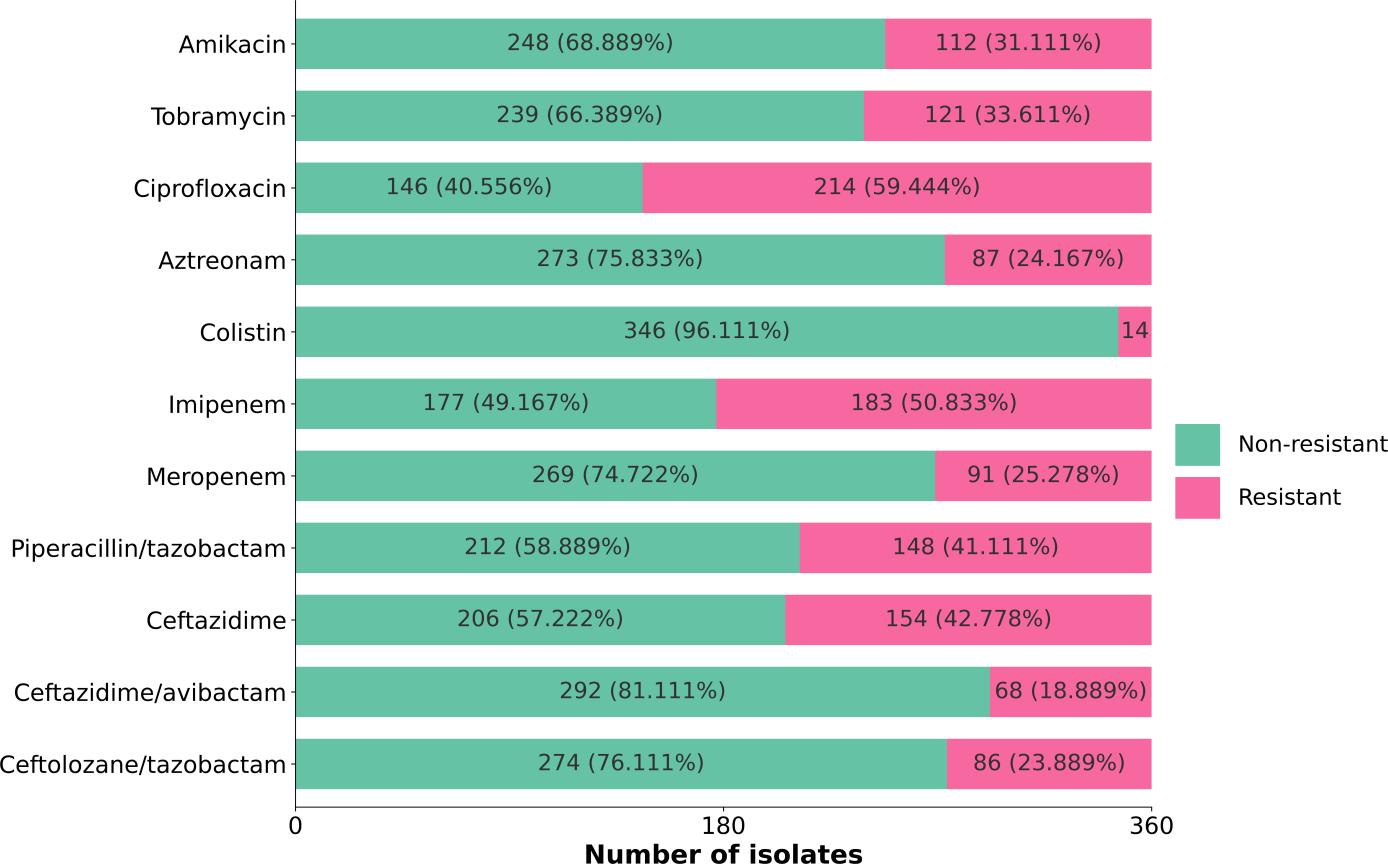
Summary of antimicrobial susceptibility testing phenotypes. Number and proportion of non-resistant and resistant isolates of each antimicrobial. Broth microdilution assays were conducted for all 360 isolates to obtain MIC values for 11 antipseudomonal agents.

We generated MALDI-TOF spectra (Bruker Daltonics) on 360 *P*. *aeruginosa* isolates from our institution collected from 2004 to 2021. Following this, we conducted phenotypic AST using broth microdilution. The resulting minimum inhibitory concentrations (MICs) for 11 anti-pseudomonal antimicrobials were categorized in binary labels as “resistant” and “non-resistant” (Fig. 1) according to established breakpoints (17).

### Developing machine learning models for MALDI-TOF MS-based AMR prediction

**Fig 2:**
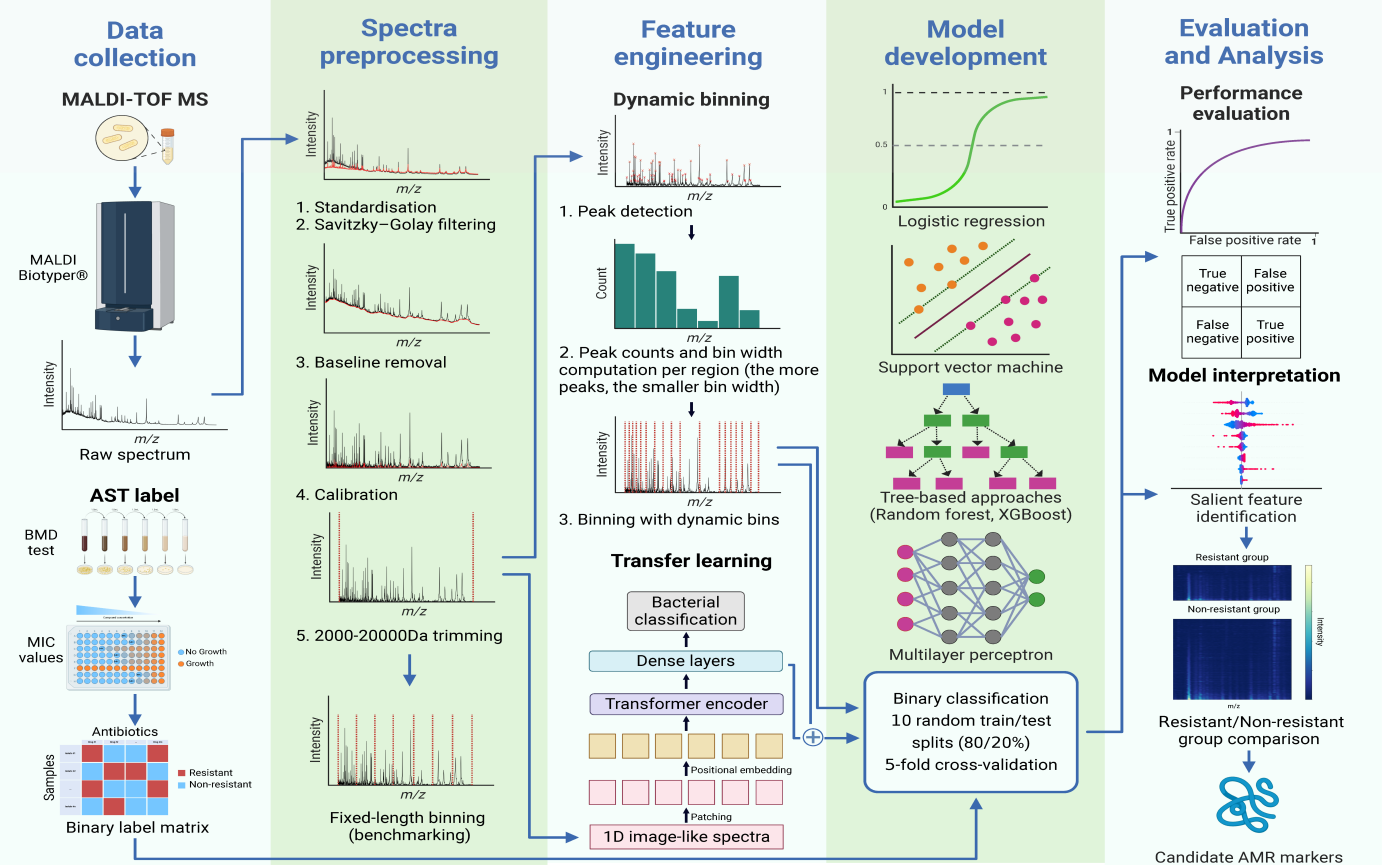
Schematic workflow of the study. Spectra preprocessing: raw spectra were preprocessed with a five-step pipeline (18). To generate the benchmarking feature sets, we conducted fixed-length binning (3 Da, 20 Da, and 30 Da) of preprocessed spectra, resulting in feature vectors of 6,000, 900, and 600 dimensions, respectively. Dynamic binning: we determined the mean number of intensity peaks in 500 Da intervals from 2,000 to 20,000 Da. The bin widths used for each region (ranging from 1 Da to 100 Da) are inversely proportional to the corresponding mean peak count of that region. Latent representation learning: a vision transformer model was trained to differentiate high-risk bacterial pathogens (20). The final hidden layer of the model was used as embedding to extract additional features from the MALDI-TOF spectra. Model development: we used 5-fold cross-validation with 80%–20% training–testing split to train models. Performance evaluation: AUROC was the key performance metric. Model interpretation: the Shapley additive explanations (SHAP) algorithm was used to identify feature bins with high importance (21). We then analyzed the regions of interest to interpret models and identify potential biological AMR determinants. Abbreviations: MALDI-TOF MS, matrix-assisted laser desorption/ionization–time of flight mass spectrometry; AST, antimicrobial susceptibility testing; BMD, broth microdilution; MIC, minimum inhibitory concentration; *m*/*z*, mass-to-charge ratio.

We developed ML models using MALDI-TOF spectra as inputs to predict phenotypic susceptibility categories for each isolate and antimicrobial combination (Fig. 2). Spectra were initially preprocessed using a previously described approach (18). Next, we introduced a novel yet simple method called dynamic binning to extract features from the preprocessed spectra. Dynamic binning addresses the shortcomings of two commonly used feature engineering methods for MALDI-TOF MS data: peak detection (i.e., using intensity peaks as features) (13, 15, 19) and fixed-length binning (i.e., using bins of pre-specified size as features) (14, 18). The former approach requires additional analysis steps for peak alignment and potentially overlooks regions without peaks, while the latter approach often generates a large feature set that greatly outnumbers sample size, thereby increasing the chances of over-fitting. With dynamic binning, we consider the quantity of intensity peaks in each region to dynamically determine the bin width for the binning process (see Materials and Methods). This allowed us to generate a 769-dimensional representation vector for each isolate.

For the prediction task, we employed five distinct supervised machine learning approaches (logistic regression [LR], random forest [RF], support vector machine [SVM], LightGBM [LGB], and multilayer perceptron [MLP]). We trained and tested our models with 10 random splits and calculated the average metric values of all runs for each model and each antimicrobial.

### Successful prediction of antimicrobial susceptibility in *P. aeruginosa* using MALDI-TOF MS

**Fig 3:**
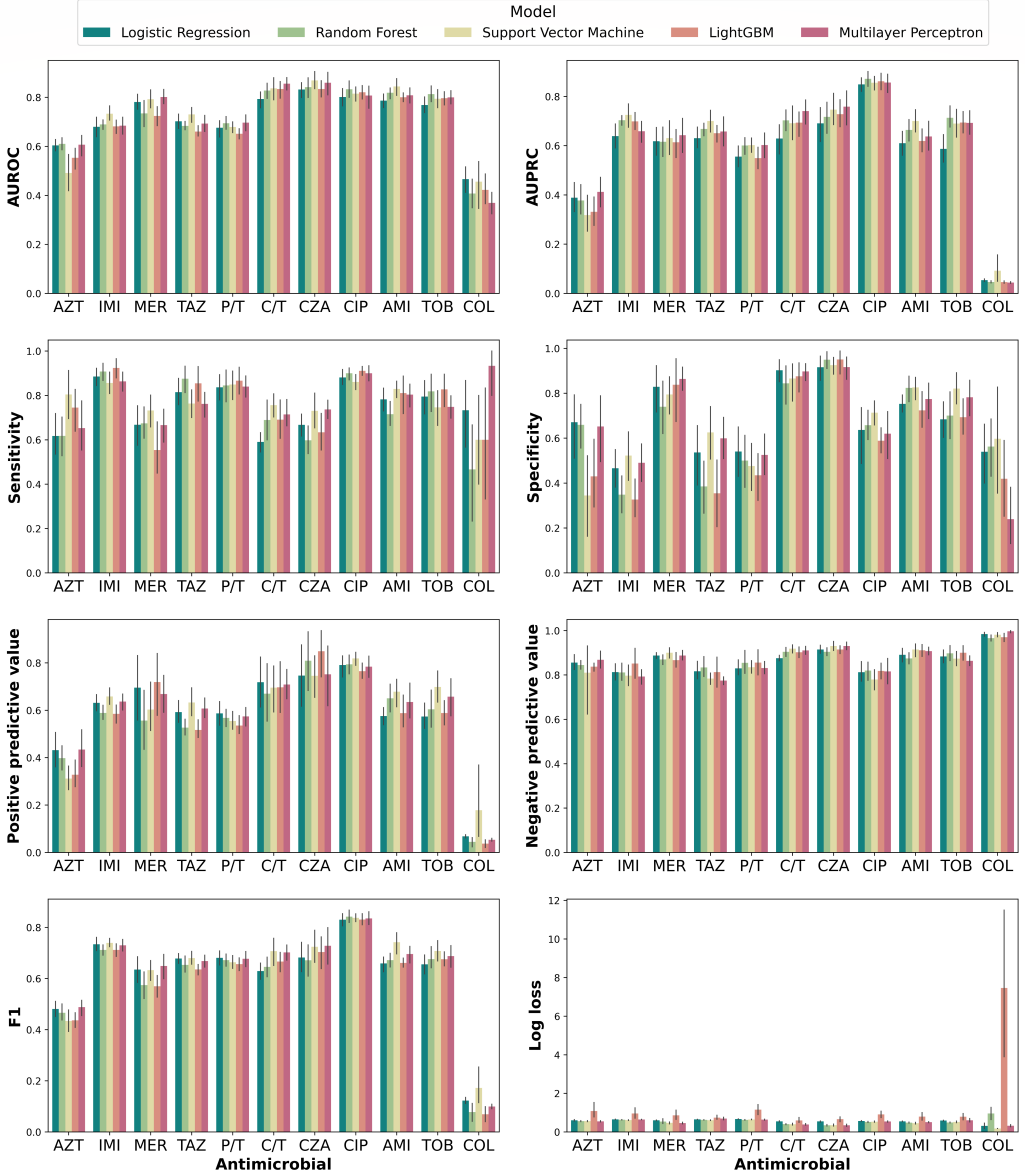
The predictive performance of dynamic binning across all tested machine learning models. The mean metric scores across 10 random splits are reported. The vertical black lines at the tip of each bar represent the 95% CI (95% CI). Abbreviations: AUROC, area under the receiver operating characteristic curve; AUPRC, area under the precision-recall curve; AZT, aztreonam; IMI, imipenem; MER, meropenem; TAZ, ceftazidime; P/T, piperacillin/tazobactam; C/T, ceftolozane/tazobactam; CZA, ceftazidime/avibactam; CIP, ciprofloxacin; AMI, amikacin; TOB, tobramycin; COL, colistin.

The predictive performance for each antimicrobial is presented in Fig. 3. In terms of area under the receiver operating characteristic curve (AUROC) performances per antimicrobial class, aminoglycosides (0.829, 95% confidence interval [CI]: 0.801–0.856) demonstrated significantly better AUROC compared to β-lactams (0.756, CI: 0.733–0.780, *P* = 0.002) and polymyxin (0.466, CI: 0.402–0.530, *P* < 0.001) (Table S1). The β-lactam group showed a wide range of performance, from 0.610 for aztreonam (CI: 0.579–0.641) to 0.869 for ceftazidime/avibactam (CI: 0.826–0.912). Notably, our models performed well for ceftolozane/tazobactam (0.856, CI: 0.824–0.887) and ceftazidime/avibactam (0.869, CI: 0.826–0.912), two novel β-lactam/β-lactamase inhibitors considered as key treatments for multidrug-resistant *P. aeruginosa* infections. This subgroup achieved higher performance than for other β-lactams (*P* < 0.001) and polymyxins (*P* < 0.001) and on par performance for fluoroquinolones (*P* = 0.155) and aminoglycosides (*P* = 0.169). The lowest performance was observed for polymyxins (colistin), possibly due to the highly imbalanced data set (only 14 of 360 resistant isolates). Regarding metrics related to clinical decision-making, the best specificity was achieved for ceftazidime/avibactam (0.925, CI: 0.878–0.972), and the best sensitivity was achieved for ciprofloxacin (0.900, CI: 0.866–0.933). The SVM algorithm most frequently achieved highest AUROC in 4 of 11 antimicrobials. This was followed by the RF and MLP algorithms, each achieving the highest AUROC in 3 of 11 antimicrobials.

### Performance assessment of dynamic binning as a feature engineering approach

We performed a comparative analysis of dynamic binning with different settings of fixed-length binning approach across 10 antimicrobials, excluding colistin due to the highly imbalanced data. For fixed-length binning, we first selected the commonly used 3 Da bin width (16, 18). For the MALDI-TOF MS spectral range of 2,000–20,000 Da, this resulted in a feature set of 6,000 features. We then chose bin widths that yielded feature vector dimensions similar to dynamic binning (*n* = 769), specifically 20 Da and 30 Da widths, resulting in feature sets of 900 and 600 features, respectively.

**Fig 4:**
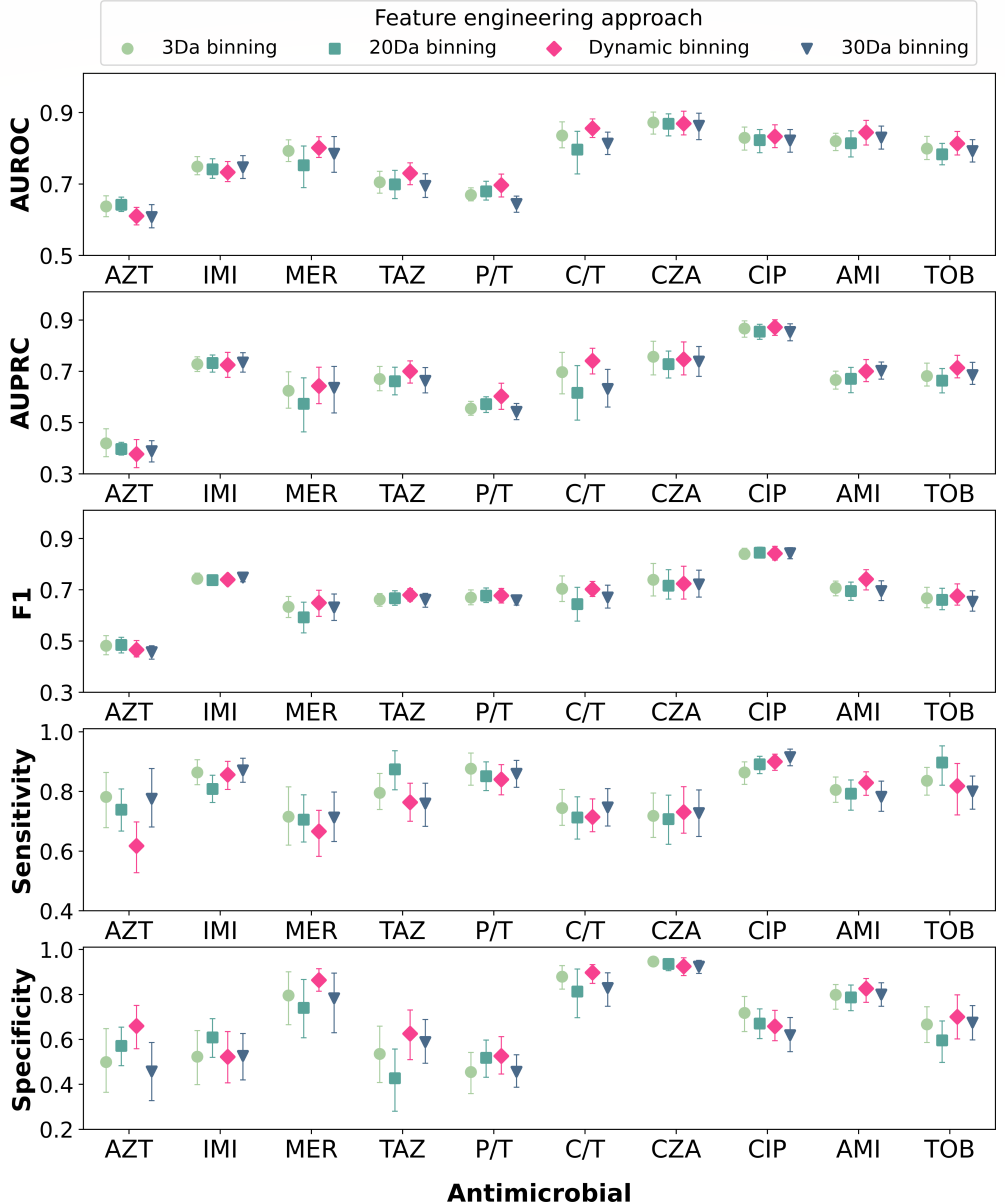
Comparison of dynamic binning and fixed-length binning approaches. The binning approaches are ordered from left to right by the size of the feature set in descending order. Markers and lines indicate mean and 95% CI of the metrics, respectively. Abbreviations: AUROC, area under the receiver operating characteristic curve; AUPRC, area under the precision-recall curve; AZT, aztreonam; IMI, imipenem; MER:, meropenem; TAZ, ceftazidime; P/T, piperacillin/tazobactam; C/T, ceftolozane/tazobactam; CZA, ceftazidime/avibactam; CIP, ciprofloxacin; AMI, amikacin; TOB, tobramycin.

Dynamic binning demonstrated the highest AUROC for 7 of 10 antimicrobials (meropenem, ceftazidime, ceftolozane/tazobactam, piperacillin/tazobactam, ciprofloxacin, amikacin, and tobramycin) and second-best AUROC for ceftazidime/avibactam (Fig. 4; Table S2). While 3 Da binning generated a much larger feature set (6,000), it achieved slightly better AUROC than dynamic binning in aztreonam (0.637 vs 0.610, *P* = 0.824), ceftazidime/avibactam (0.872 vs 0.869, *P* = 0.862), and imipenem (0.749 vs 0.733, *P* = 0.824). Compared to conventional binning approaches with similar number of features, dynamic binning outperformed 20 Da binning and 30 Da binning in 7 of 10 and in 9 of 10 antimicrobials, respectively. The largest AUROC improvements were observed in ceftolozane/tazobactam (7.5%) in comparison with 20 Da binning and in piperacillin/tazobactam (8.2%) in comparison with 30 Da binning. This finding suggests that conventional binning strategies may not capture the complex patterns in the MALDI-TOF MS data associated with AMR in *P. aeruginosa*, highlighting the advantages of dynamic binning in effectively capturing relevant information while maintaining a compact feature set size and thus reducing computational burden.

### Machine learning approach using dynamic binning achieved high performance in an external data set

We validated our approach using the DRIAMS data set, an external MALDI-TOF MS data set with 4,139 *P*. *aeruginosa* spectra with corresponding AST data (18). In contrast to our data set, AST was performed using various methods (VITEK 2, bioMérieux; MIC Test Strips, Liofilchem; disc diffusion, ThermoFisher), and average spectral intensity values were lower (Fig. S1). In terms of AST label (Table S3), the Alfred data set had significantly higher resistance rates than the DRIAMS data set for seven of eight tested antimicrobials (*P* < 0.001, χ test).

**Fig 5:**
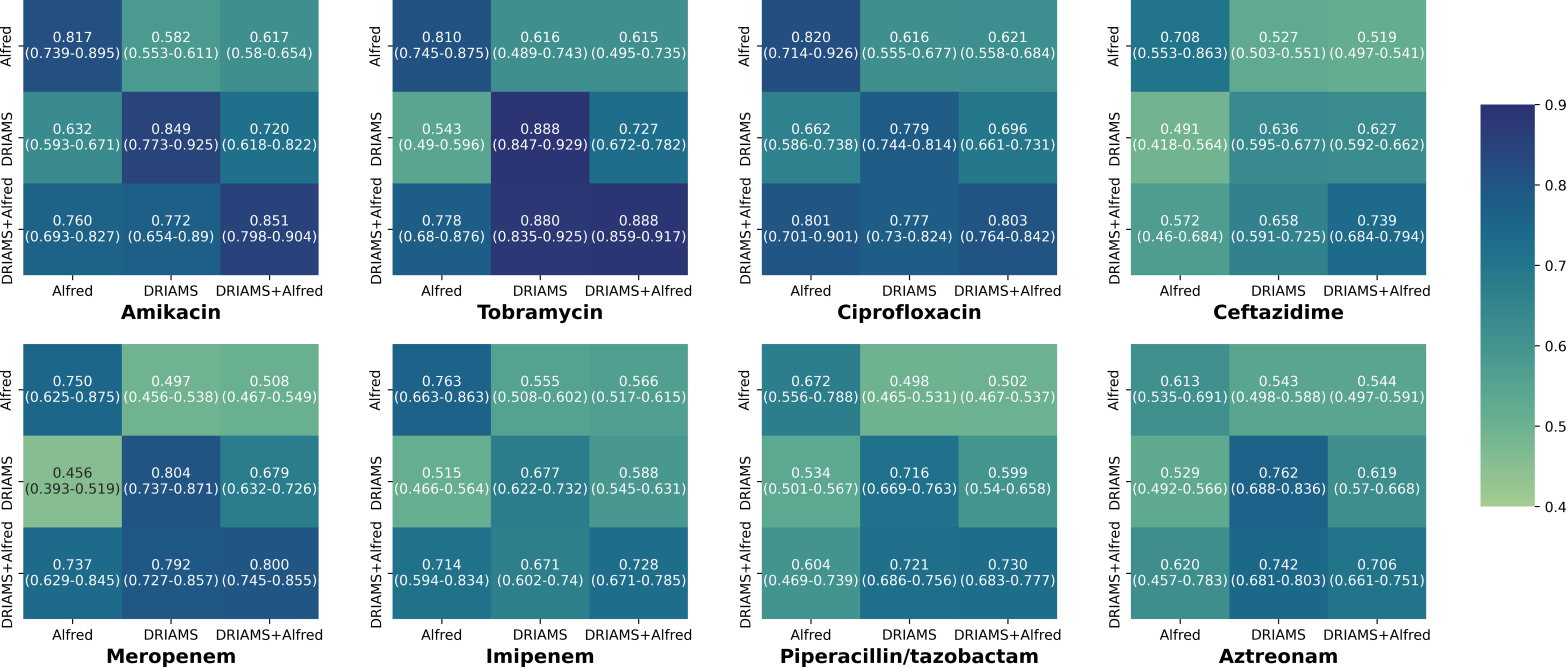
Cross-institution performance of dynamic binning. Different training (*y*-axis) and testing (*x*-axis) sets using DRIAMS and Alfred Hospital data were used to train and evaluate the models. Data are reported as mean AUROC (95% CI) across 10 random splits. Abbreviations: AUROC, area under the receiver operating characteristic curve; DRIAMS, Database of Resistance Information on Antimicrobials and MALDI-TOF Mass Spectra.

With these caveats in mind, we used dynamic binning and the same methodology described above to train ML models for the eight antimicrobials with AST labels from both the Alfred and DRIAMS data sets (Fig. 5). Different combinations of Alfred and DRIAMS data were used in the training and testing data sets. Dynamic binning demonstrated efficacy for the DRIAMS data set, with AUROC values ranging from 0.636 (CI: 0.595–0677) in ceftazidime to 0.888 (CI: 0.847–0.929) in tobramycin. Notably, while there was no statistically significant difference in AUROC between dynamic binning and 3 Da binning approaches for all tested antimicrobials, the training time for models trained with dynamic binning features was significantly shorter for six of eight antimicrobials (Table S4). Across all training/testing set scenarios, our models consistently performed significantly better with internal isolates compared to external isolates (*P* < 0.001), suggesting the presence of overfitting. We noted that using additional data from external institutions in the training set could adversely affect internal performance (Table S5). For instance, in the case of the Alfred test set, the addition of DRIAMS data significantly reduced AUROC for ceftazidime (*P* = 0.018). Although the same effect was observed in five of seven antimicrobials in the DRIAMS test set, the difference was only statistically significant for amikacin (*P* = 0.036), possibly due to the large sample size of the DRIAMS training set.

### Further improvement in MALDI-TOF MS predictions through transfer learning

Transfer learning has been widely adopted in multiple fields of ML. Transfer learning uses knowledge from one domain/task to improve performance in a related domain/task (22). In brief, rather than training a model *de novo*, a model previously trained on a large data set is used as a starting point. This pre-trained model is then fine-tuned or used as an embedding layer for a new task using a specific data set relevant to this new task.

In order to retain key information after dimensionality reduction with dynamic binning, we used transfer learning to retrieve a compressed embedding of spectral data and used this to engineer additional features. Specifically, a vision transformer was used to develop a classification model for bacterial species (incorporating *>*20,000 spectra) (see Materials and Methods) (20). The model achieved a testing accuracy of 0.98, not only with the internal testing set but also with external data (Fig. S2).

We applied this embedding to *P. aeruginosa* spectra, generating additional 256 features for each sample. We then used a stacking method to combine the learning outcomes from the dynamic binning features and the features obtained from the latent representation (see Materials and Methods). When compared to baseline models using only dynamic binning features, integrating transfer learning showed higher AUROC values in 8 of 11 antimicrobials but did not reach statistical significance (Table 1). The largest change was observed for meropenem, with an AUROC of 0.844 compared to 0.801 for the baseline model.

**TABLE 1:** AUROC performance of dynamic binning, linear probing, and stacking approaches^*a*^

Antimicrobial	Dynamic binning	Linear probing	Stacking	*P*
Aztreonam	0.610 (CI: 0.579–0.641)	0.600 (CI: 0.540–0.661)	0.588 (CI: 0.542–0.634)	0.607
Imipenem	0.733 (CI: 0.698–0.768)	0.543 (CI: 0.504–0.583)	**0.740 (CI: 0.708–0.771**)	0.715
Meropenem	0.801 (CI: 0.765–0.838)	0.529 (CI: 0.479–0.578)	**0.844 (CI: 0.800–0.887**)	0.607
Ceftazidime	0.730 (CI: 0.693–0.767)	0.533 (CI: 0.483–0.582)	0.712 (CI: 0.675–0.75)	0.607
Piperacillin/tazobactam	0.697 (CI: 0.658–0.735)	0.585 (CI: 0.523–0.647)	**0.716 (CI: 0.688–0.745**)	0.607
Ceftolozane/tazobactam	0.856 (CI: 0.824–0.887)	0.553 (CI: 0.508–0.598)	**0.876 (CI: 0.829–0.923**)	0.607
Ceftazidime/avibactam	0.869 (CI: 0.826–0.912)	0.515 (CI: 0.46–0.570)	**0.891 (CI: 0.849–0.934**)	0.607
Ciprofloxacin	0.833 (CI: 0.793–0.873)	0.526 (CI: 0.486–0.566)	**0.848 (CI: 0.814–0.881**)	0.607
Amikacin	0.844 (CI: 0.802–0.887)	0.524 (CI: 0.463–0.586)	0.839 (CI: 0.814–0.865)	0.607
Tobramycin	0.813 (CI: 0.773–0.853)	0.525 (CI: 0.463–0.587)	**0.823 (CI: 0.776–0.869**)	0.607
Colistin	0.466 (CI: 0.402–0.53)	0.645 (CI: 0.528–0.762)	**0.487 (CI: 0.381–0.592**)	0.94

^*a*^ Results are presented as mean and 95% CI. Bold text indicates when stacking model achieved better results. We report the statistical testing results (*P*-values) comparing the stacking and dynamic binning approaches.

### Identifying potential AMR biomarkers with dynamic binning

**Fig 6:**
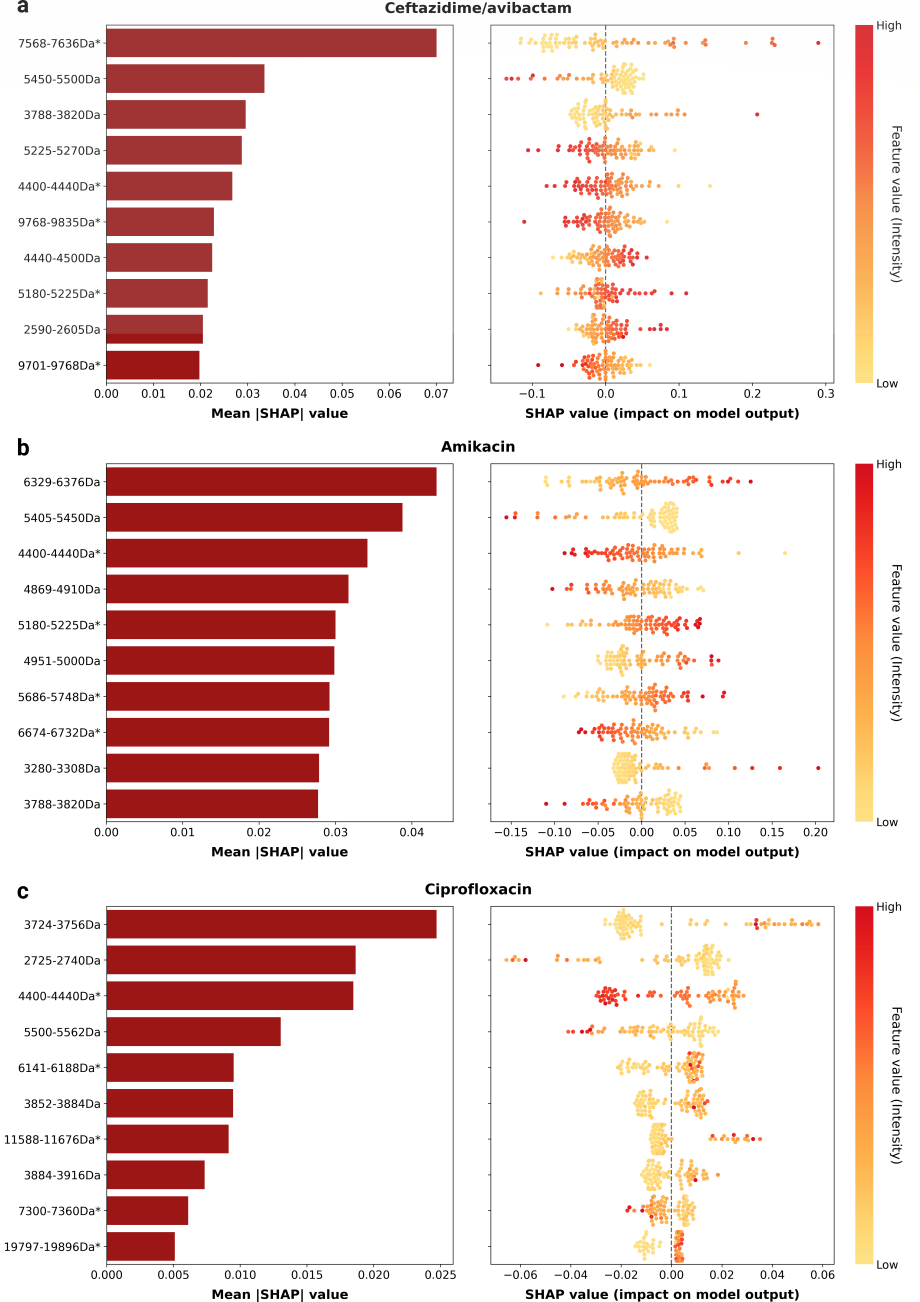
Model interpretation analysis using the SHAP algorithm for (**a**) ceftazidime/avibactam, (**b**) ciprofloxacin, and (**c**) tobramycin. The left panel for each antimicrobial displays the top 10 contributing features in descending order of the mean absolute SHAP value (from top to bottom). The *y*-axis labels include the spectral ranges, with the asterisks indicating the presence of reviewed proteins within the region. The right panels display the SHAP value of each individual sample for the corresponding features on the left. Each sample dot is color-coded by the magnitude of the feature value.

Interpretability of ML models is a key aspect of facilitating their future implementation in healthcare applications. To conduct feature importance analysis, we utilized the SHAP algorithm to compute the contribution of each feature in the best ML algorithm for three representatives achieving the highest AUROC performance in three antimicrobial classes: ceftazidime/avibactam (β-lactams), amikacin (aminoglycosides), and ciprofloxacin (fluoroquinolones) (Fig. 6) (21). The spectral ranges with the highest contribution in these antimicrobials were 7,568–7,636 Da, 6,329–6,376 Da, and 3,724–3,756 Da, respectively. In all three antimicrobials, high signal intensity within these spectral regions contributed to the prediction of resistance. Consistent with Weis's observation (18), most top 10 contributing features were located in regions < 10,000 Da (all for ceftazidime/avibactam and amikacin, and 8 of 10 in ciprofloxacin). We conducted a mapping with the UniProt database and identified a list of reviewed proteins and the number of unreviewed proteins present within the top contributing feature bins (Table S6).

In the region contributing the most to ceftazidime/avibactam resistance (7,568–7,636 Da), resistant isolates displayed a unimodal distribution, with 72.1% exhibiting a peak at 7,600–7,620 Da. Conversely, non-resistant isolates demonstrated a bimodal distribution, with peaks at 7,600–7,620 Da (45.890%) and 7,570–7,590 Da (20.548%) (Fig. S3). This difference resulted in a significantly lower mean peak intensity (*P* = 2.3 × 10^−5^) in the 7,600–7,620 Da region for non-resistant isolates. Three reviewed proteins, namely, protein RegB, major cold shock protein CspA, and UPF0337 protein PA4738, were identified within the feature bin 7,568–7,636 Da.

Using the same approach, we investigated the most important spectral ranges in amikacin and ciprofloxacin. In ciprofloxacin-resistant isolates, a significantly increased signal intensity was observed in the region 3,735–3,745 Da (*P* = 6.3 × 10^−9^) within the feature bin of 3,724–3,756 Da (Fig. S4). In the case of amikacin, we did not observe any significant difference between resistant and non-resistant isolates for the feature bin of 6,329–6,376 Da. Although no reviewed proteins were found, we identified 12 and 58 unreviewed proteins mapping to the ciprofloxacin and amikacin regions, respectively (Table S6).

## DISCUSSION

In this study, we successfully used novel machine learning methods to predict AMR for *P. aeruginosa* from MALDI-TOF MS data. Despite the complexity of resistance mechanisms in *P. aeruginosa*, we achieved high predictive performance using five different ML approaches across 11 antimicrobials, including novel β-lactam/β-lactamase inhibitor agents. We also showed validity of our approach with a comparable high performance using *P. aeruginosa* MALDI-TOF MS data from a publicly available data set. MALDI-TOF MS data are now routinely generated in clinical microbiology for bacterial identification testing, and our work demonstrates that these data could be successfully re-purposed for rapidly inferring AST without additional costs.

The performance of our models varied depending on the antimicrobial type (AUROC of 0.466–0.869), consistent with previous studies using MALDI-TOF MS to predict AMR (15, 16, 18). Our models performed well for fluoroquinolones and aminoglycosides (AUROC of 0.813–0.844), which aligns with previous findings, using not only MALDI-TOF MS but also genomic data (15, 23–26). In studies utilizing genomic data, machine learning models not only achieved good predictive performance but were also able to identify canonical AMR genes related to these antimicrobials, such as gyrA and parC for fluoroquinolones (23, 25) and aminoglycoside-modifying enzymes (AMEs) for tobramycin (27). This suggests that the effect of these resistance mechanisms is strong enough to confer AMR (28), and the machine learning models are able to detect them effectively. In contrast, β-lactams showed more variable performance, reflecting the complexity of AMR mechanisms. This has also been noted in *Klebsiella pneumoniae*, with AUROC for carbapenems ranging from 0.55 to 0.95 (18, 29). Nguyen et al*.* observed similar results using genomic data to predict AMR in *K. pneumoniae*, achieving high accuracy in MIC prediction for both fluoroquinolones (0.97–0.98) and aminoglycosides (0.95–0.97), while having more mixed performance for β-lactams (0.61–1.00) (27). The heterogeneity of these findings highlights the challenge in developing a reliable AMR prediction model using MALDI-TOF MS.

In comparison to AMR prediction performance of models using whole genome sequencing (WGS) data (25), our approach using MALDI-TOF MS data and dynamic binning achieved superior AUROC for two of three tested antimicrobials: amikacin (0.844 vs 0.795) and meropenem (0.801 vs 0.790). For ceftazidime, the WGS-based model achieved better performance (0.730 vs 0.882). This finding is highly encouraging, considering the lower cost and labor associated with obtaining MALDI-TOF spectra compared to genomic data (30). Integration of multiple data modalities may potentially improve performance and highlight a possible future direction for enhancing AMR prediction models. For instance, Khaledi et al*.* effectively combined genomic with transcriptomic data to predict AMR in *P. aeruginosa*, achieving F1-macro scores of 0.82–0.92 (23).

The varying performance of MALDI-TOF MS AMR predictions across antimicrobial classes observed in our study has important clinical implications. For ciprofloxacin, MALDI-TOF MS models could be used as a rule-out test for resistance due to their high sensitivity (0.900). This may help prevent treatment failure in outpatient settings, as ciprofloxacin is one of the few oral treatments available for *P. aeruginosa* infections. On the other hand, the high specificity observed in ceftazidime/avibactam (0.925) and ceftolozane/tazobactam (0.897) can be used as a rule-in test for resistance. Confirmed resistance to these antimicrobials could prompt clinicians to consider alternative treatment options. In addition, we found that our models maintain good positive predictive values for ceftazidime/avibactam (0.745) and ceftolozane/tazobactam (0.708) at relatively low resistance rates (18.9% and 23.9%, respectively).

Our benchmarking analysis demonstrated that dynamic binning and transfer learning may improve performance. Compared to the conventional feature engineering approach, dynamic binning performed on par or better in 7 of 10 tested antimicrobials. These approaches complement each other: dynamic binning reduces irrelevant features, while transfer learning adds potentially useful ones. Healthcare-related data often require feature reduction due to their high dimensionality and limited sample size (31). However, using a wide bin width in fixed-length binning may inadvertently lead to the loss of informative intensity peaks when multiple peaks fall within the same bin. This likely explains why 20 Da binning and 30 Da binning performed less effectively than 3 Da binning. A recent study used the DRIAMS data set to predict AMR in *P. aeruginosa* with deep learning models and 3 Da binning (6,000 features) (16). Despite using fewer features (769 features), our dynamic binning approach achieved comparable performance and outperformed a sub-analysis in that study using a selected set of 1,000 features. This highlights the efficacy of our dynamic binning approach in preserving the predictive performance compared to feature selection methods.

The improvements associated with transfer learning were modest, but models can benefit from incorporating informative features. Although our pre-trained model showed excellent performance in bacterial identification (the primary use case of MALDI-TOF MS in clinical microbiology settings), this task may be relatively easy and not sufficiently informative for subsequent use in AMR prediction. To effectively apply transfer learning for AMR prediction, further work is needed, such as tailoring specific pre-trained models or collecting more diverse data sets (32). Alternative pre-training tasks have been reported. For instance, De Waele et al. employed a self-supervised approach using a masked language model to predict the intensity of masked peaks before fine-tuning the pre-trained model for AMR prediction (33). Regarding the latter approach, Visonà et al. demonstrated a proof-of-concept that incorporating data from multiple species can help improve AMR prediction in a specific species (34).

We demonstrated the effectiveness of dynamic binning by validating it on a large public data set. Our models generally performed better when the training and testing sets were from the same site, consistent with Weis et al.'s findings (18). Interestingly, Yu et al. noted that their model, which predicts methicillin-resistant *Staphylococcus aureus*, performed better on prospective data from their center than data sets from other healthcare centers (13). This may be explained by underlying biological differences in geographically different data sets (35), such as clonal relatedness and differences in AST profiles. Cuénod et al. demonstrated that spectral variability might be attributable to different protocols for sample preparation or device calibration across different healthcare centers (36, 37). While including additional training data can improve model performance, incorporating an external data set may introduce noise and degrade model performance. Interestingly, the collection year of samples can also affect model performance. Weis et al. showed that adding spectra from samples collected from more recent periods can help improve the predictive performance of the model (18). Therefore, to ensure the robustness of the AMR prediction models, it is crucial to repeatedly reevaluate the training process, considering the dynamic changes in pathogen characteristics over time.

In this study, we used the SHAP algorithm for post-hoc interpretability. Our findings indicate that the presence or absence of peaks significantly influences the model's performance, consistent with previous work (13, 18, 29). The proteins RegB, CspA, and UPF0337 protein PA4738 were identified within the most contributing bin of our best performing model for predicting ceftazidime/avibactam resistance. RegB is known to facilitate production of exotoxin A, a potent virulence factor in *P. aeruginosa* (38). The presence of CspA is correlated with survival rates in extreme weather environments (39). The PA4738 gene encodes a hydrophilin protein, which is expressed under conditions of osmotic stress (40). While we were able to identify salient biomarkers that show potential relationships to AMR mechanisms, interpreting these findings proved to be challenging for several reasons. Firstly, there are limited data available on the changes in MALDI-TOF spectra that are specifically related to AMR in *P. aeruginosa*. Additionally, the large numbers of uncharacterized proteins further complicate the interpretation of key features. Literature-based manual curation requires substantial time and effort, contrasting with the rapid generation of numerous newly identified protein entities through high-throughput methods (41). Consequently, the biomarkers identified through model interpretation analysis should be considered as hypothesis-generating rather than confirmed AMR determinants. For example, while limited research has been conducted on the effect of hydrophilin proteins on AMR in *P. aeruginosa*, preliminary results have demonstrated their association with antimicrobial tolerance in this species (42).

We acknowledge several limitations. The small size of our data set made it challenging to apply cutting-edge deep learning architectures, which typically require a larger training data set. However, combining our data set with publicly available data did not necessarily improve performance, suggesting that further work in building specific deep learning models for MALDI-TOF data is needed. Furthermore, obtaining WGS data on all isolates was beyond the scope of our study but may have allowed us to better understand the population structure of tested isolates and identify genetic resistance mechanisms. While we identified putative resistance determinants through model interpretation, these need experimental validation (43). In our study, multiple proteins had similar masses, making it difficult to identify exact AMR biomarkers. This problem could be overcome by molecular docking simulations to analyze changes in antimicrobial binding affinity (13) or incorporating genomic data (44). We also acknowledge that some resistance determinants may have a mass that is outside the spectral range used for bacterial identification (approximately 2,000–20,000 Da) (45). However, resistance may potentially be inferred through detection of surrogate biomarkers. For example, the mecA protein that confers methicillin resistance in *S. aureus* has a mass of 28,311 Da, but the presence of a smaller product, PSM-mec, at 2,415 Da can be used to confirm mecA-carrying *S. aureus* isolates (46). Lastly, the translation of this work to clinical settings will require rigorous prospective evaluation for antimicrobials that achieved good performance such as ciprofloxacin, ceftazidime/avibactam, or ceftolozane/tazobactam. Current efforts have been limited to retrospective studies that evaluate potential deleterious outcomes resulting from disagreements between clinical decisions and machine learning model predictions. However, Weis et al. found that, in cases where the classifier-guided antimicrobial prescription differed from the empiric antimicrobial regimen, 89% of patients would potentially benefit from the change suggested by the models (18). Additionally, Yu et al. demonstrated that antimicrobial adjustment based on AMR prediction results could lower the mortality rate in patients with carbapenem-resistant *K. pneumoniae* infection (29).

In conclusion, our study provides key insights into how AI approaches can successfully predict AMR in *P. aeruginosa* across multiple antimicrobial classes from MALDI-TOF MS data. Our findings demonstrate that model performance can be improved through efficient feature engineering techniques, such as dynamic binning, and by leveraging extensive data sets. Future directions include evaluating dynamic binning with other high-risk pathogens and integrating multiple data modalities, not only to improve predictive performance but also to foster explainable AI.

## MATERIALS AND METHODS

### MALDI-TOF MS data collection

The study was reviewed and approved by the Alfred Hospital Ethics Committee (Project No. 185/21). We systematically reviewed all *P. aeruginosa* isolates in an institutional collection from 2004 to 2021 at the Alfred Hospital (Melbourne, Australia) and conducted MALDI-TOF analysis. The Alfred Hospital is a 638-bed quaternary hospital, with a cystic fibrosis and lung transplant state referral service. The spectra were acquired from clinical samples collected from patients, including blood, urine, respiratory tract, and wound specimens. We excluded all spectra that were not identified as *P. aeruginosa* by MALDI-TOF. For isolates collected from the same patient with an identical AST pattern, we randomly selected a representative isolate to include in the data set. The final data set comprises 360 *P*. *aeruginosa* isolates.

MALDI-TOF MS acquisition was performed with the Microflex LT MALDI-TOF System (Bruker Daltonik GmbH) on bacterial colony smears prepared using the Extended Direct Transfer method in accordance with the manufacturer's instructions. Briefly, a thin smear was prepared from well isolated colonies on a MALDI 96 MPS steel target plate, and the dried smear was overlaid with 70% formic acid, followed by HCCA solution (Bruker α-cyano-4-hydroxycinnamic acid [HCCA] matrix dissolved in standard solvent containing 50% acetonitrile and 2.5% trifluoroacetic acid). The air-dried preparations were analyzed by Microflex LT MALDI-TOF System operated in a standard linear ionization mode, accelerating voltage: 20 kV, nitrogen laser frequency: 60 Hz, and 240 laser shots to generate raw data within an *m*/*z* ratio between 2,000 and 20,000 Da.

### Antimicrobial susceptibility testing

We conducted AST for all isolates by performing broth microdilution using the Sensititre (ThermoFisher) Gram-negative plate (plate code: DKMGN). The reported MIC values were then converted to binary labels using the European Committee on Antimicrobial Susceptibility Testing version 13 (EUCAST v13.0) breakpoint tables (17). Specifically, we classified isolate as resistant (1) if the MIC value is greater than (*>*) the resistant breakpoint and as non-resistant (0) if the MIC value is less than or equal to (≤) the resistant breakpoint.

### Spectra preprocessing

We followed the five-step workflow outlined in reference 18. In brief, the pipeline includes (i) variance stabilization with square root transformation, (ii) data smoothing with the Savitzky-Golay filter, (iii) baseline removal with SNIP, (iv) total-ion-current (TIC) calibration, and (v) 2,000–20,000 Da range trimming. Preprocessed spectra will be binned with either dynamic binning or fixed-length binning (Fig. 2). We explored the use of wider bin widths in order to reduce the number of features used in fixed-length binning and thus approximate the number of dynamic binning features, as previously reported (47). Specifically, we chose the bin widths of 20 Da (900 features) and 30 Da (600 features). This resulted in feature sets with sizes both above and below the number of features generated by the dynamic binning method.

### Dynamic binning method

Firstly, we computed the average peak counts for 500 Da disjoint sub-regions. With the preprocessed spectra, we used MALDIquant to detect intensity peaks (48). For each sample, we counted the number of peaks appearing in each sub-region and finally calculated the average number of peaks across the whole data set. Secondly, we determined a bin width value, ranging from 1 to 100 Da, for each sub-region, which is inversely proportional to its corresponding average peak count. In contrast to the binning process in reference 18, which used mean values, we computed the maximum value of each bin. This was done as bin widths in dynamic binning can be much larger than in fixed-length binning, making it possible for a high intensity value to be diluted out by a large number of low intensity values if a mean is used. Following both dynamic binning and fixed-length binning, we obtained a tabular data representation where each row represents a sample and the columns represent the maximum intensity of each bin.

### Machine learning model development

Firstly, the data set was randomly split into a training (80%) and testing (20%) set. For each antimicrobial, we used the same 10 train and test sets for all tested models to ensure a fair comparison across the different models evaluated. We then used Scikit-learn and its related packages to build our models (49, 50). We used the following machine learning approaches: LR, RF, SVM, LGB, and MLP with hyperparameter tuning to select the best performing hyperparameter set for each model. Specifically, we followed a nested cross-validation approach to tune hyperparameters. We used 5-fold cross-validation on the training set as the inner loop to optimize the model hyperparameters based on AUROC. Here, we used Scikit-learn's StratifiedKFold to evenly distribute susceptibility labels into the training and validation sets. The best performing model from the inner loop was then tested on the held-out test set in the outer loop, with this process repeated over 10 iterations. For each tunable hyperparameter, we used Optuna to determine the optimized value after 100 trials. Based on the history of the trial records, Optuna used a Bayesian approach, namely, Tree-structured Parzen Estimator, to decide the next value to assess (50). The list of hyperparameters was adapted from reference 18 and can be found in Table S7. To account for an imbalanced data set, we implemented a class weight setting that adjusts the contribution of each class to the loss function during the training process. We followed approach of Weis et al. to keep this setting as a tunable parameter (18). This approach downweighs the output of the majority class and outweighs the output of the minority class when computing loss values. The class weights are inversely proportional to the sample ratio between resistant and non-resistant groups.

### Performance analysis and statistical testing

For performance evaluation, we predicted AMR for the testing set using the fine-tuned models. The classification threshold was chosen to maximize the F1-score. We selected AUROC as our primary performance metric. Additionally, we reported the area under the precision-recall curve (AUPRC), F1-score, sensitivity, specificity, positive predictive value (PPV), negative predictive value (NPV), and log-loss (51, 52). These metrics are defined as follows:


$$sensitivity=recall=\;\frac{true\;positive}{true\;positive+false\;negative}$$

$$specificity=\;\frac{true\;negative}{true\;negative+false\;positive}$$

$$precision=\;\frac{true\;positive}{true\;positive+false\;positive}$$

$$PPV=\;\frac{true\;positive}{true\;positive+false\;positive}$$

$$NPV=\;\frac{true\;negative}{true\;negative+false\;negative}\;$$

$$F1=\;2\;\times \frac{precision\;\times \;recall}{precision+recall}$$

log loss= −[ylog⁡(p)+(1−y)log(1−p)]

where *y* is the ground truth label (0: non-resistant, 1: resistant) and *p* is the predicted probability. Here, we used the natural logarithm. Unless otherwise specified, all metrics are presented as mean and 95% CI values. We used Kruskal-Wallis H test to compare performance across different settings (53). For false discovery control, the *P*-values of multiple comparisons were adjusted by using Benjamini-Hochberg procedure (54).

### External data set assessment

We identified 4,139 *P*. *aeruginosa* isolates in the publicly available DRIAMS data set (18). These isolates had both MALDI-TOF MS spectra and AST data for eight antimicrobials: amikacin, tobramycin, ciprofloxacin, ceftazidime, meropenem, imipenem, piperacillin/tazobactam, and aztreonam. We followed the same process as described above to preprocess the raw spectra, extract features, and train and evaluate the models. For each antimicrobial, we assessed three permutations using different data sets (only Alfred Hospital data, only DRIAMS data, and all data) for training and testing, resulting in nine different settings for each antimicrobial. Since different versions of the EUCAST tables (v6-9) were used to convert the AST data in DRIAMS data set, we reconciled the labels from EUCAST v6-9 to EUCAST v13 to ensure a uniform labeling system. We noted that, for tobramycin, isolates previously classified as tobramycin-susceptible according to the old EUCAST versions (susceptible: ≤4 mg/L; resistant: >4 mg/L) can now be classified as tobramycin-resistant based on the updated EUCAST v13 (susceptible: ≤2 mg/L; resistant: >2 mg/L). Therefore, we have not discussed the performance difference between the internal and external test sets for tobramycin. For all other antimicrobials, the “S” (susceptible) and “R” (resistant) labels in the previous breakpoint references were classified into the non-resistant and resistant groups, respectively. For antimicrobials with an “I” (intermediate) category, we reviewed the breakpoint tables across all versions and either re-classified them into the resistant group (e.g., ciprofloxacin, imipenem) or the non-resistant group (e.g., amikacin, meropenem, aztreonam) to maintain consistency in the labeling.

### Bacterial identification with vision transformer

We built a vision transformer model to classify high-risk pathogens, including *Enterobacter cloacae*, *Staphylococcus aureus*, *Klebsiella pneumoniae*, *Acinetobacter baumannii*, *Pseudomonas aeruginosa*, and *Enterococcus faecium*. Each spectrum was viewed as a one-dimensional image. To enhance the resolution, we used 1 Da binning features, resulting in an input shape of (1, 1, 18,000). We then split the spectra into 1,000 smaller patches using a patch size of (1, 18). These patches were then linearly embedded before being fed into a standard transformer model. The vision transformer model consisted of eight attention heads, and the final hidden layer has 256 units. We used cross entropy loss as the loss function, and the models were trained for 1,000 epochs with a batch size of 128, learning rate of 10^−3^, and dropout rate of 0.2. The model from the best checkpoint, determined via validation loss, was selected as the final model. We used Pytorch and Pytorch-lightning for the training and testing process (55, 56). For latent representation visualization, we used t-SNE to project the embedding into two-dimensional space (57).

### AMR prediction with stacking method

With stacking ensemble learning, the predicted outputs generated by multiple base estimators are used as features for the final estimator (Fig. S5). Here, we utilized Scikit-learn's StackingClassifier to develop the stacking model, with LR, RF, SVM, LGB, and MLP as the base estimators. For the final meta-learning estimator, we experimented with all the aforementioned algorithms and selected the final stacking model that achieved the highest AUROC.

Instead of learning from the entire combined feature set, we created two distinct base models for each ML approach. One model learns from the dynamic binning features, while the other learns from the latent embedding features. This approach results in a total of 10 different features for each sample, allowing the stacking model to leverage the strengths of both feature engineering techniques and potentially capture more diverse patterns in the data.

### Model interpretation with SHAP algorithms

We utilized the “shap” library to analyze feature contributions for the best performing model per antimicrobial (21). The algorithm computes the contributing score of each individual feature by measuring the difference in the model's performance when the corresponding feature is included or excluded in different permutations of feature sets. We used TreeExplainer for RF and LGB, as well as KernelExplainer for LR, SVM and MLP (21, 58). To handle possible multicollinearity of input features, we used the “interventional” option (59). We mapped top 10 highest contributing *m*/*z* ranges to the UniProt database (https://www.uniprot.org) to identify potential AMR biomarkers. To ensure relevance, we only selected proteins related to *P. aeruginosa*. The list of proteins and their corresponding masses were obtained using UniProt's search and download interface.

## Data Availability

The data sets and computer code produced in this study are available in the following databases: MALDI-TOF spectra and AST data, Mendeley (DOI: 10.17632/tpcznh9968.110.17632/tpcznh9968.1) (60); source code, GitHub (https://github.com/andyvng/pae-amr-maldi) (61).

## SUPPLEMENTAL MATERIAL

Supplemental File msystems.00789-24-s0001.docx Supplemental figures and tables.

ASM does not own the copyrights to Supplemental Material that may be linked to, or accessed through, an article. The authors have granted ASM a non-exclusive, world-wide license to publish the Supplemental Material files. Please contact the corresponding author directly for reuse.
