# Supplementary material for: Predicting Pseudomonas aeruginosa drug resistance using artificial intelligence and clinical MALDI-TOF mass spectra
Source: mSystems. 2024 Aug 16;9(9):e00789-24. doi: 10.1128/msystems.00789-24 (PMC11406958; doi:10.1128/msystems.00789-24)
Supplement: Supplemental File — Supplemental figures and tables. [file msystems.00789-24-s0001.docx]

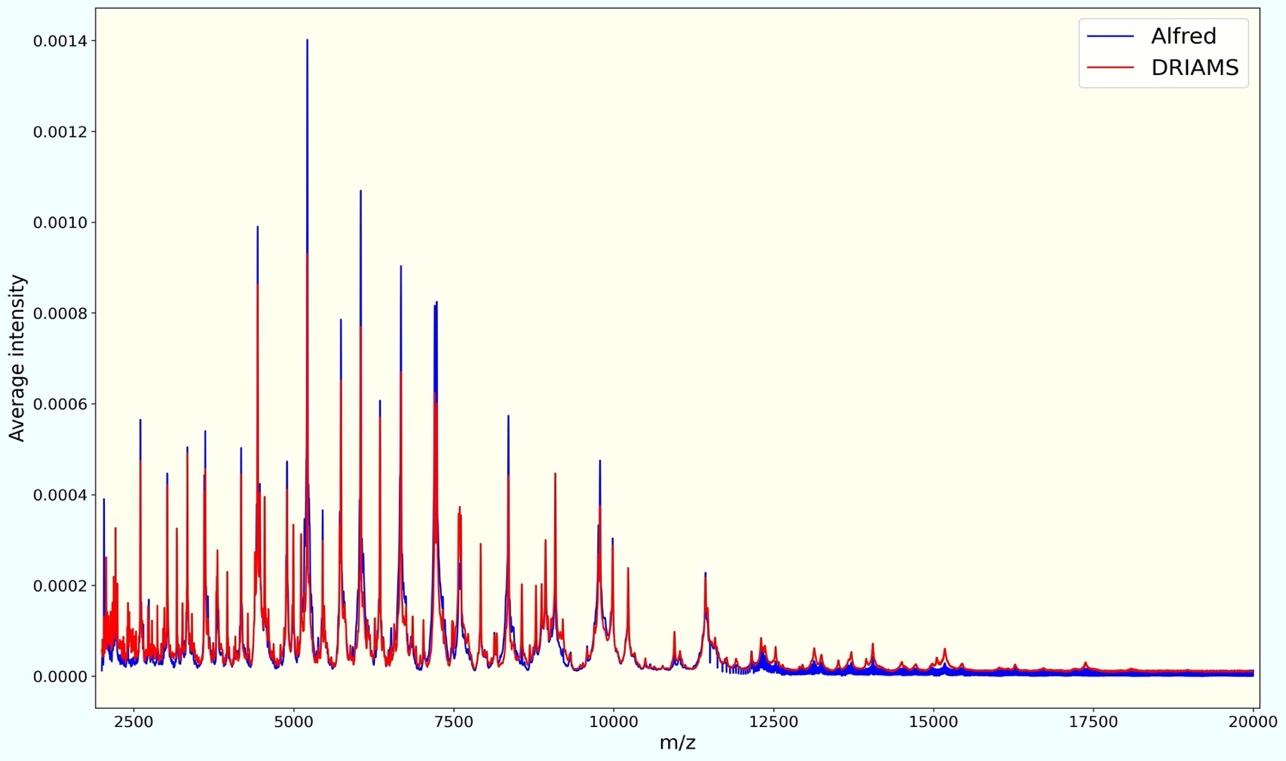


**Supplementary Fig. 1 - Differences in peak intensity between DRIAMS and Alfred Hospital datasets.**  Mean intensity values of MALDI-TOF spectra of P. aeruginosa from the DRIAMS and Alfred Hospital datasets are shown. All MALDI-TOF spectra were preprocessed and trimmed to 2,000-20,000 Da. We used 1-Da binning to generate a uniformed intensity vector of 18,000 dimensions for each isolate.

Abbreviations: DRIAMS - Database of Resistance Information on Antimicrobials and MALDI-TOF Mass Spectra; m/z – mass-to-charge ratio


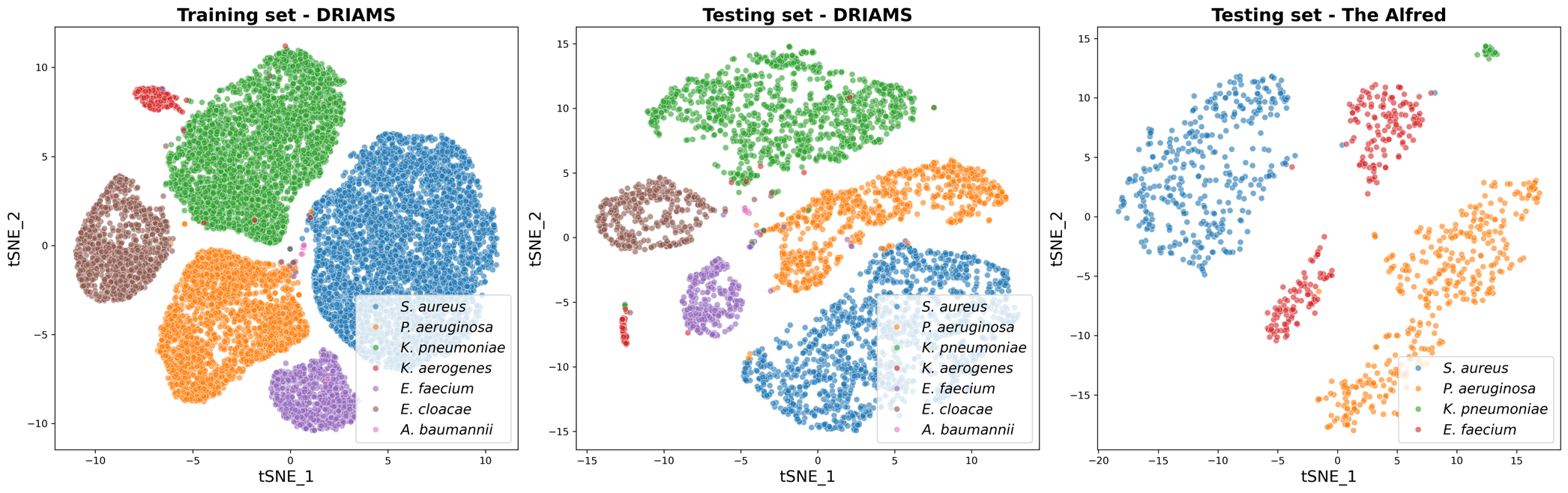


**Supplementary Fig. 2 - Visualization of latent representation.** We used t-distributed Stochastic Neighbor Embedding (t-SNE) to project the hidden layer retrieved from the vision transformer model into 2-D space. Shown are the clusters of pathogens in the training set (left, DRIAMS dataset), testing set (center, DRIAMS dataset), and Alfred Hospital dataset (right).

Abbreviations: DRIAMS - Database of Resistance Information on Antimicrobials and MALDI-TOF Mass Spectra; tSNE - t-distributed Stochastic Neighbor Embedding; *S. aureus* – *Staphylococcus aureus*; *P. aeruginosa* – *Pseudomonas aeruginosa*; *K. pneumoniae* – *Klebsiella pneumoniae*; *E. faecium* – *Enterococcus faecium*; *E. cloacae* – *Enterobacter cloacae*; *K. aerogenes - Klebsiella aerogenes; A. baumannii* – *Acinetobacter baumannii.*


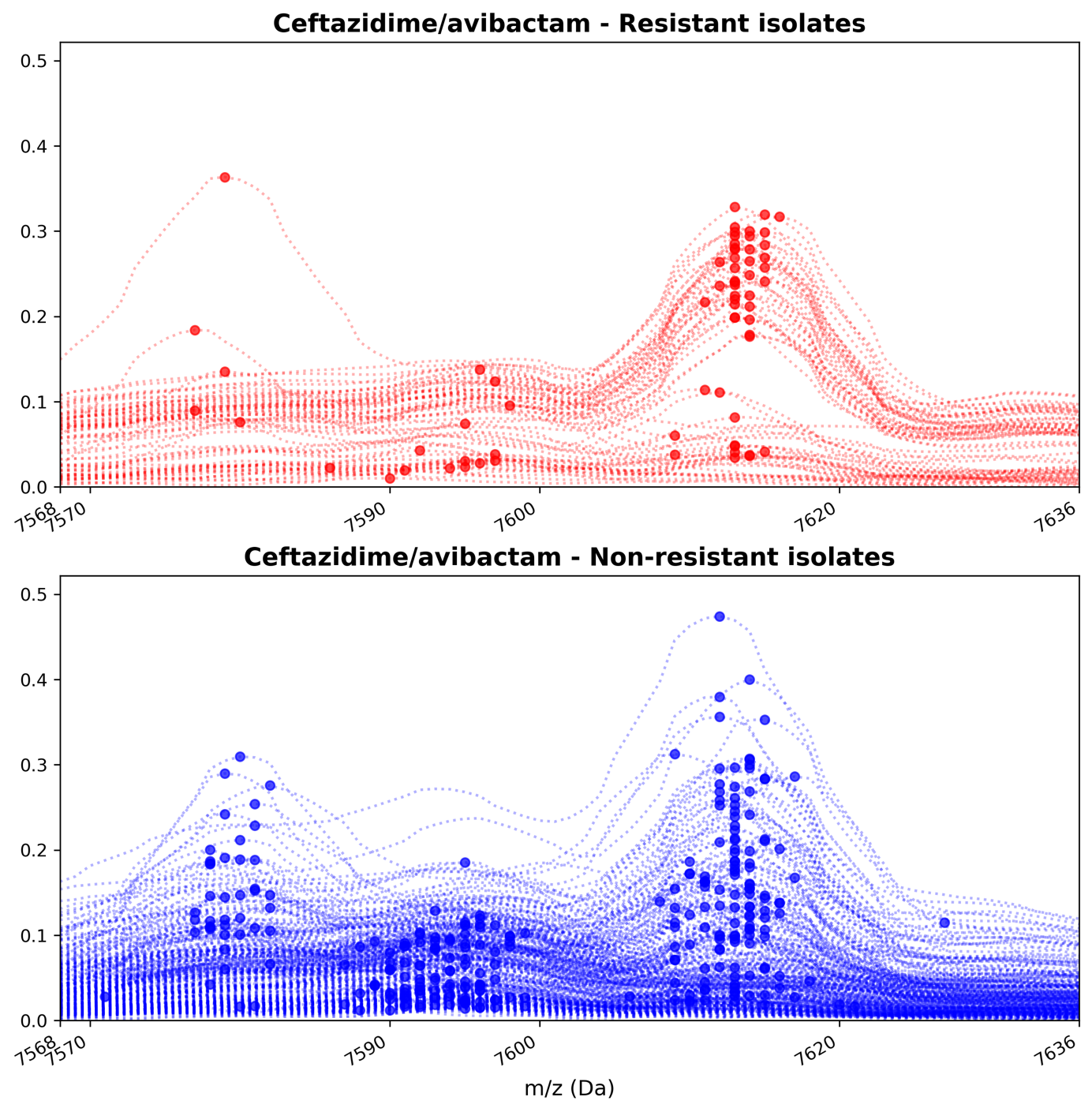


**Supplementary Fig. 3 – MALDI-TOF spectra of cetazidime/avibactam resistant and non-resistant isolates within the most contributing bin (7568-7636 Da).**

**
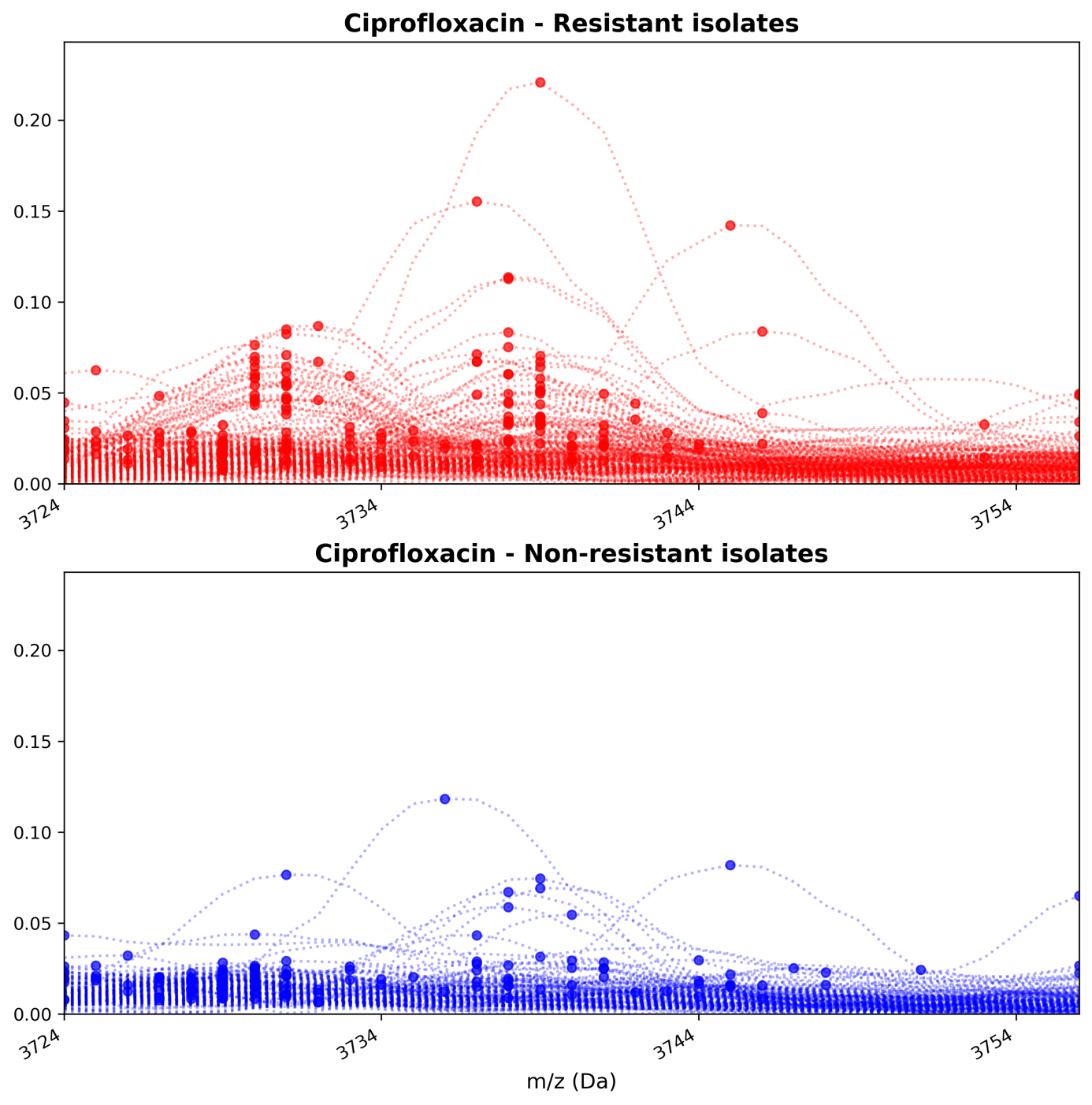
**

**Supplementary Fig. 4 - MALDI-TOF spectra of ciprofloxacin resistant and non-resistant isolates within the most contributing bin (3724-3756 Da).**


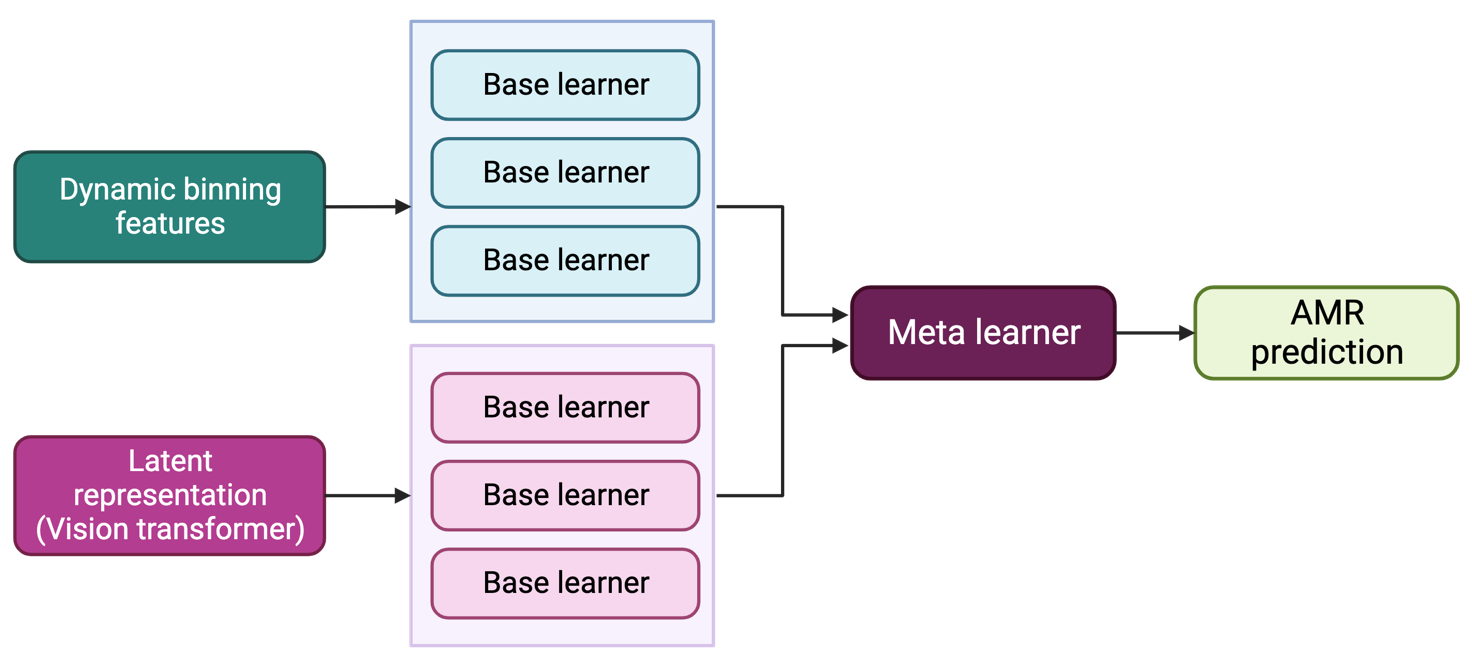


**Supplementary Fig. 5 - High-level architecture of stacking model.** Each input data type, i.e., dynamic binning features and latent representation from vision transformer model, was trained separately with different base learners.

Abbreviations: AMR – antimicrobial resistance.

**Supplementary Table 1 - Pairwise AUROC performance comparison between antimicrobial classes.** Data is presented as mean and 95% CI.

| **Group 1** | **Group 2** | **AUROC (Group 1)** | **AUROC (Group 2)** | **P (adjusted)** |
| --- | --- | --- | --- | --- |
| Aminoglycosides | β-lactams | 0.829 (CI: 0.801-0.856) | 0.756 (CI: 0.733-0.78) | 0.002 |
| Aminoglycosides | Fluoroquinolones | 0.829 (CI: 0.801-0.856) | 0.833 (CI: 0.793-0.873) | 0.930 |
| Aminoglycosides | Polymyxins | 0.829 (CI: 0.801-0.856) | 0.466 (CI: 0.402-0.530) | <0.001 |
| β-lactams | Fluoroquinolones | 0.756 (CI: 0.733-0.780) | 0.833 (CI: 0.793-0.873) | 0.024 |
| β-lactams | Polymyxins | 0.756 (CI: 0.733-0.780) | 0.466 (CI: 0.402-0.530) | <0.001 |
| Fluoroquinolones | Polymyxins | 0.833 (CI: 0.793-0.873) | 0.466 (CI: 0.402-0.530) | <0.001 |
| Novel β-lactam/β-lactamase inhibitors | Aminoglycosides | 0.862 (CI: 0.838-0.886) | 0.829 (CI: 0.801-0.856) | 0.169 |
| Novel β-lactam/β-lactamase inhibitors | Fluoroquinolones | 0.862 (CI: 0.838-0.886) | 0.833 (CI: 0.793-0.873) | 0.155 |
| Novel β-lactam/β-lactamase inhibitors | Other β-lactams | 0.862 (CI: 0.838-0.886) | 0.714 (CI: 0.692-0.737) | <0.001 |
| Novel β-lactam/β-lactamase inhibitors | Polymyxins | 0.862 (CI: 0.838-0.886) | 0.466 (CI: 0.402-0.530) | <0.001 |

**Supplementary Table 2 - Pairwise AUROC performance comparison between dynamic binning and conventional binning approaches.** Data is presented as mean and 95% CI.

| **Antimicrobial** | **Dynamic binning** | **3-Da binning** | | **20-Da binning** | | **30-Da binning** | |
| --- | --- | --- | --- | --- | --- | --- | --- |
|  |  | **AUROC** | ***P*** | **AUROC** | ***P*** | **AUROC** | ***P*** |
| Meropenem | 0.801 (CI 0.765-0.838) | 0.793 (CI 0.755-0.830) | 0.862 | 0.753 (CI 0.683-0.822) | 0.597 | 0.785 (CI 0.723-0.846) | 0.870 |
| Amikacin | 0.844 (CI 0.802-0.887) | 0.820 (CI 0.790-0.850) | 0.824 | 0.814 (CI 0.769-0.859) | 0.597 | 0.83 (CI 0.791-0.869) | 0.802 |
| Aztreonam | 0.610 (CI 0.579-0.641) | 0.637 (CI 0.601-0.673) | 0.824 | 0.642 (CI 0.616-0.667) | 0.530 | 0.607 (CI 0.567-0.648) | 0.870 |
| Ciprofloxacin | 0.833 (CI 0.793-0.873) | 0.829 (CI 0.787-0.871) | 0.94 | 0.823 (CI 0.785-0.861) | 0.821 | 0.822 (CI 0.784-0.86) | 0.870 |
| Piperacillin/tazobactam | 0.697 (CI 0.658-0.735) | 0.669 (CI 0.646-0.692) | 0.824 | 0.679 (CI 0.649-0.710) | 0.597 | 0.644 (CI 0.616-0.672) | 0.210 |
| Ceftolozane/tazobactam | 0.856 (CI 0.824-0.887) | 0.835 (CI 0.791-0.880) | 0.824 | 0.796 (CI 0.726-0.867) | 0.530 | 0.813 (CI 0.774-0.852) | 0.452 |
| Colistin | 0.466 (CI 0.402-0.530) | 0.457 (CI 0.351-0.562) | 0.870 | 0.433 (CI 0.341-0.525) | 0.667 | 0.438 (CI 0.351-0.524) | 0.870 |
| Tobramycin | 0.813 (CI 0.773-0.853) | 0.799 (CI 0.760-0.838) | 0.857 | 0.783 (CI 0.747-0.819) | 0.553 | 0.792 (CI 0.754-0.830) | 0.706 |
| Ceftazidime | 0.730 (CI 0.693-0.767) | 0.705 (CI 0.666-0.743) | 0.824 | 0.699 (CI 0.650-0.747) | 0.637 | 0.695 (CI 0.653-0.736) | 0.479 |
| Ceftazidime/avibactam | 0.869 (CI 0.826-0.912) | 0.872 (CI 0.834-0.911) | 0.862 | 0.869 (CI 0.830-0.908) | 0.776 | 0.863 (CI 0.816-0.910) | 0.940 |
| Imipenem | 0.733 (CI 0.698-0.768) | 0.749 (CI 0.716-0.781) | 0.824 | 0.741 (CI 0.710-0.773) | 0.667 | 0.747 (CI 0.709-0.785) | 0.870 |

**Supplementary Table 3 - Comparison of resistance rate between The Alfred and DRIAMS datasets (Chi-square test).** Data were collected from multiple healthcare centers using different antimicrobial susceptibility testing assay methods. This resulted in varying numbers of isolates tested for each antimicrobial.

| **Antimicrobial** | **The Alfred** | **DRIAMS** | **P (Adjusted)** |
| --- | --- | --- | --- |
| Amikacin | 112/360 (31.111%) | 106/3141 (3.375%) | < 0.001 |
| Tobramycin | 121/360 (33.611%) | 258/3489 (7.395%) | < 0.001 |
| Ciprofloxacin | 214/360 (59.444%) | 740/3962 (18.677%) | < 0.001 |
| Ceftazidime | 154/360 (42.778%) | 293/3281 (8.930%) | < 0.001 |
| Meropenem | 91/360 (25.278%) | 336/3554 (9.454%) | < 0.001 |
| Imipenem | 183/360 (50.833%) | 420/3170 (13.249%) | < 0.001 |
| Piperacillin/tazobactam | 148/360 (41.111%) | 676/3965 (17.049%) | < 0.001 |
| Aztreonam | 87/360 (24.167%) | 212/987 (21.479%) | 0.329 |

**Supplementary Table 4** – **Comparison of AUROC and training time between dynamic binning vs 3-Da binning on DRIAMS dataset.** Data are presented as mean and 95%CI.

| **Antimicrobial** | **AUROC** | | | **Training time (hours)** | | |
| --- | --- | --- | --- | --- | --- | --- |
|  | **Dynamic binning** | **3-Da binning** | ***P*** | **Dynamic binning** | **3-Da binning** | ***P*** |
| Meropenem | 0.804  (CI: 0.738-0.871) | 0.820  (CI: 0.756-0.885) | 0.603 | 0.158  (CI: 0.138-0.179) | 1.509  (CI: 1.123-1.895) | <0.001 |
| Imipenem | 0.677  (CI: 0.623-0.732) | 0.690  (CI: 0.634-0.745) | 0.603 | 12.577  (CI: 3.941-21.213) | 17.136  (CI: 3.404-30.868) | 0.149 |
| Ceftazidime | 0.636  (CI: 0.595-0.677) | 0.639  (CI: 0.573-0.704) | 0.970 | 0.149  (CI: 0.114-0.183) | 1.470  (CI: 1.103-1.837) | <0.001 |
| Piperacillin/tazobactam | 0.716  (CI: 0.669-0.762) | 0.747  (CI: 0.695-0.798) | 0.124 | 1.452  (CI: 0.507-2.397) | 10.496  (CI: 8.067-12.925) | <0.001 |
| Aztreonam | 0.762  (CI: 0.688-0.836) | 0.761  (CI: 0.696-0.826) | 0.970 | 2.064  (CI: 0.138-3.991) | 2.326  (CI: 0.428-4.223) | 0.364 |
| Ciprofloxacin | 0.779  (CI: 0.743-0.815) | 0.777  (CI: 0.745-0.809) | 0.970 | 10.301  (CI: 6.268-14.335) | 27.725  (CI: 15.097-40.352) | <0.001 |
| Amikacin | 0.849  (CI: 0.773-0.924) | 0.866  (CI: 0.784-0.948) | 0.649 | 0.196  (CI: 0.166-0.226) | 2.623  (CI: 1.874-3.371) | <0.001 |
| Tobramycin | 0.888  (CI: 0.847-0.929) | 0.896  (CI: 0.839-0.952) | 0.649 | 0.149  (CI: 0.113-0.186) | 1.358  (CI: 0.989-1.728) | <0.001 |

**Supplementary Table 5. AUROC performance comparison between internal training set (The Alfred or DRIAMS) and combined training set (The Alfred and DRIAMS).** Data are presented as mean AUROC. The 95% CI values can be found in Fig. 5.

| **Antimicrobial** | **Test set** | **Internal training set** | **Combined training set** | ***P*** |
| --- | --- | --- | --- | --- |
| Aztreonam | DRIAMS | 0.762 | 0.742 | 0.482 |
| Imipenem | DRIAMS | 0.677 | 0.671 | 1.000 |
| Meropenem | DRIAMS | 0.805 | 0.793 | 0.795 |
| Ceftazidime | DRIAMS | 0.636 | 0.658 | 0.453 |
| Piperacillin/tazobactam | DRIAMS | 0.716 | 0.721 | 0.795 |
| Ciprofloxacin | DRIAMS | 0.779 | 0.777 | 1.000 |
| Amikacin | DRIAMS | 0.849 | 0.773 | 0.036 |
| Aztreonam | The Alfred | 0.613 | 0.620 | 1.000 |
| Imipenem | The Alfred | 0.763 | 0.714 | 0.201 |
| Meropenem | The Alfred | 0.750 | 0.737 | 0.800 |
| Ceftazidime | The Alfred | 0.709 | 0.572 | 0.018 |
| Piperacillin/tazobactam | The Alfred | 0.672 | 0.604 | 0.113 |
| Ciprofloxacin | The Alfred | 0.820 | 0.801 | 0.615 |
| Amikacin | The Alfred | 0.817 | 0.760 | 0.054 |

**Supplementary Table 6. Mapping result of the most contributing feature bins to Uniprot’s database in three representative antimicrobials.** Here, we show details of reviewed proteins and the number of unreviewed proteins for each bin.

| **Antimicrobial** | **Bin (m/z)** | **Gene (Entry)** | **Number of unreviewed proteins** |
| --- | --- | --- | --- |
| Ceftazidime/avibactam | 7568-7636Da | regB (Q03381), cspA (P95459), PA4738 (Q9HV61) | 201 |
|  | 5450-5500Da | N/A | 54 |
|  | 3788-3820Da | N/A | 14 |
|  | 5225-5270Da | N/A | 43 |
|  | 4400-4440Da | rpmJ (Q9HWF6), rpmJ1 (Q02T59) | 39 |
|  | 9768-9835Da | rpoZ (B7V5M8), minE (B7V7X4), PLES_32771 (B7VA59), PA14_38060 (Q02LJ2), PA2045 (Q9I270) | 153 |
|  | 4440-4500Da | N/A | 61 |
|  | 5180-5225Da | rpmH (A6VF47) | 34 |
|  | 2590-2605Da | N/A | 1 |
|  | 9701-9768Da | ptsH (Q9HVV2) | 158 |
| Amikacin | 6329-6376Da | N/A | 58 |
|  | 5405-5450Da | N/A | 33 |
|  | 4400-4440Da | rpmJ (Q9HWF6), rpmJ1 (Q02T59) | 39 |
|  | 4869-4910Da | N/A | 44 |
|  | 5180-5225Da | rpmH (A6VF47) | 34 |
|  | 4951-5000Da | N/A | 43 |
|  | 5686-5748Da | PA0567 (Q9I5W9) | 74 |
|  | 6674-6732Da | PSPA7_2181 (A6V3B8), PLES_20821 (B7V148), PA14_25520 (Q02PE3), PA2980 (Q9HZM4), ccmD (Q9I3N4) | 84 |
|  | 3280-3308Da | N/A | 6 |
|  | 3788-3820Da | N/A | 14 |
| Ciprofloxacin | 3724-3756Da | N/A | 12 |
|  | 2725-2740Da | N/A | 1 |
|  | 4400-4440Da | rpmJ (Q9HWF6), rpmJ1 (Q02T59) | 39 |
|  | 5500-5562Da | N/A | 47 |
|  | 6141-6188Da | rubA2 (Q9HTK8) | 53 |
|  | 3852-3884Da | N/A | 17 |
|  | 11588-11676Da | rplU (B7V0B1), zapA (Q9HTW3), PA4753 (P95453) | 243 |
|  | 3884-3916Da | N/A | 12 |
|  | 7300-7360Da | pscE (Q9I317) | 90 |
|  | 19797-19896Da | apt (Q02K26) | 172 |

**Supplementary Table 7. List of tuning parameters for each model.**

| Model | Parameter (Scikit-learn) | Settings (Optuna) |
| --- | --- | --- |
| Logistic Regression | tol | [1e-6, 1e-3], log=True |
|  | penalty | L2 |
|  | c | [1e-2, 1], log=True |
|  | max_iter | [500, 1000], step=100 |
|  | class_weight | [None, balance] |
| Random forest | n_estimators | [100, 1000], step=100 |
|  | max_depth | [1, 29], step=2 |
|  | max_features | [20, 800], step=20 |
|  | criterion | [gini, entropy, log_loss] |
|  | bootstrap | [True, False] |
|  | class_weight | [None, balance, balanced_subsample] |
| Support vector machine | c | [1e-5, 1e5], log=True |
|  | kernel | [linear, poly, rbf] |
|  | gamma | [scale, auto] |
|  | class_weight | [None, balance] |
| LightGBM | boosting_type | [gdbt, dart, goss] |
|  | n_estimators | [25, 2000], step=25 |
|  | learning_rate | [1e-5, 1e4], log=True |
|  | class_weight | [None, balance] |
| Multi-layer perceptron | hidden_layer_sizes | (256, 128, 64) |
|  | solver | [sgd, adam] |
|  | momentum | [0, 1] |
|  | max_iter | [200, 1000], step=100 |
|  | learning_rate_init | [1e-5, 1e-3], log=True |
|  | activation | [tanh, relu] |
|  | power_t | [0.2, 0.8], step=0.1 |
